# Supplementary material for: Ontogeny shapes individual dietary specialization in female European brown bears (Ursus arctos)
Source: Nat Commun. 2024 Nov 29;15:10406. doi: 10.1038/s41467-024-54722-z (PMC11607087; doi:10.1038/s41467-024-54722-z)
Supplement: Supplementary file 3 — Reporting Summary [file 41467_2024_54722_MOESM3_ESM.pdf]

nature portfolio

Corresponding author(s): Anne G Hertel

Last updated by author(s): Nov 5, 2024

Reporting Summary

Nature Portfolio wishes to improve the reproducibility of the work that we publish. This form provides structure for consistency and transparency in reporting. For further information on Nature Portfolio policies, see our Editorial Policies and the Editorial Policy Checklist.

Statistics

For all statistical analyses, confirm that the following items are present in the figure legend, table legend, main text, or Methods section.

n/a	Confirmed	
☐	☒	The exact sample size (*n*) for each experimental group/condition, given as a discrete number and unit of measurement
☐	☒	A statement on whether measurements were taken from distinct samples or whether the same sample was measured repeatedly
☐	☒	The statistical test(s) used AND whether they are one- or two-sided<br>*Only common tests should be described solely by name; describe more complex techniques in the Methods section.*
☐	☒	A description of all covariates tested
☐	☒	A description of any assumptions or corrections, such as tests of normality and adjustment for multiple comparisons
☐	☒	A full description of the statistical parameters including central tendency (e.g. means) or other basic estimates (e.g. regression coefficient) AND variation (e.g. standard deviation) or associated estimates of uncertainty (e.g. confidence intervals)
☐	☒	For null hypothesis testing, the test statistic (e.g. *F*, *t*, *r*) with confidence intervals, effect sizes, degrees of freedom and *P* value noted<br>*Give P values as exact values whenever suitable.*
☐	☒	For Bayesian analysis, information on the choice of priors and Markov chain Monte Carlo settings
☐	☒	For hierarchical and complex designs, identification of the appropriate level for tests and full reporting of outcomes
☐	☒	Estimates of effect sizes (e.g. Cohen's *d*, Pearson's *r*), indicating how they were calculated

Our web collection on statistics for biologists contains articles on many of the points above.

Software and code

Policy information about availability of computer code

Data collection	No software was used to collect data.
Data analysis	We used the Open source software R version 4.4.1 to prepare and analyze all data. R packages used are mentioned in the methods section of the manuscript and in the open access code available under: https://osf.io/68b9u/<br><br>We used the following R packages:<br>ctmm 1.2.0 for home range calculation<br>brms 2.22.0 for all statistical analyses<br>tidyverse_2.0.0 for data handling<br>ggplot2_3.5.1 for plotting<br>pedigree_1.4.2 for handling the genetic pedigree<br>patchwork_1.3.0 for figure assembly

For manuscripts utilizing custom algorithms or software that are central to the research but not yet described in published literature, software must be made available to editors and reviewers. We strongly encourage code deposition in a community repository (e.g. GitHub). See the Nature Portfolio guidelines for submitting code & software for further information.

Data

Policy information about availability of data

All manuscripts must include a data availability statement. This statement should provide the following information, where applicable:

- Accession codes, unique identifiers, or web links for publicly available datasets
- A description of any restrictions on data availability
- For clinical datasets or third party data, please ensure that the statement adheres to our policy

The primary data generated in this study have been provided in the OSF repository under accession code https://doi.org/10.17605/OSF.IO/68B9U 78. The raw GPS & VHF location data are available under restricted access for sensitivity reasons, access can be obtained from JK through correspondence with the first author. Source data are provided with this paper.

Research involving human participants, their data, or biological material

Policy information about studies with human participants or human data. See also policy information about sex, gender (identity/presentation), and sexual orientation and race, ethnicity and racism.

Reporting on sex and gender	n/a
Reporting on race, ethnicity, or other socially relevant groupings	n/a
Population characteristics	n/a
Recruitment	n/a
Ethics oversight	n/a

Note that full information on the approval of the study protocol must also be provided in the manuscript.

Field-specific reporting

Please select the one below that is the best fit for your research. If you are not sure, read the appropriate sections before making your selection.

☐ Life sciences ☐ Behavioural & social sciences ☒ Ecological, evolutionary & environmental sciences

For a reference copy of the document with all sections, see nature.com/documents/nr-reporting-summary-flat.pdf

Ecological, evolutionary & environmental sciences study design

All studies must disclose on these points even when the disclosure is negative.

Study description

We here use repeated individual annual estimates of bear diet, derived from stable isotopes deposited in bear hair samples taken during captures to study individual dietary specialization. Specifically, we determine the sources of dietary specialization (measured as trophic position, see below under research sample) as age, social maternal learning, environmental composition on the home range scale, genetic heritability, and indirect maternal effects. For this we composed a multi generational dataset of known mother - daughter pairs with repeated measures of trophic position, known age, known home range location after independence, occurrence in the populations genetic pedigree. First we fitted a linear mixed effects model with daughter trophic position as response variable age as fixed effect and a random intercept for individual identity. We determined how much of the total phenotypic variance could be explained by among-individual variance, i.e. "dietary specialization". Second, we used a spatially explicit Bayesian hierarchical model (i.e. 'animal model') to partition among-individual variance in trophic position into environmental similarity ($\sigma 2env$), additive genetic ($\sigma 2a$), permanent among-individual ($\sigma 2ind$), maternal ($\sigma 2mat$), and residual within-individual effects ($\sigma 2r$). We tested for maternal learning of offspring trophic position by incorporating the mother's trophic position as a covariate into the model. To account for a potential decrease of the maternal learning effect over time, we let maternal trophic position interact with the time since separation of mother and daughter (both scaled by their standard deviation and centered). We estimated the variance explained by the respective two components of the fixed effect (age and maternal learning).

All models were fit using the R package "brms". We ran four chains which were run for 6,000 iterations, with a warmup of 3,000 iterations and a thinning interval of 10. All estimated model coefficients and credible intervals were therefore based on 1200 posterior samples and had satisfactory convergence diagnostics with $\hat{R} < 1.01$, and effective sample sizes > 400. Posterior predictive checks recreated the underlying Gaussian distribution of trophic position well. For all parameters, we report the median and 89% credible intervals, calculated as equal tail intervals, as measure of centrality and uncertainty. We deemed explained variance proportions as inconclusive when the lower credible interval limit was < 0.001 (i.e., < 0.1%). All statistical analyses were performed in R 4.0.0 81. Primary data and code to reproduce all analyses are provided under (https://doi.org/10.17605/OSF.IO/68B9U).

Research sample

Hair samples were collected from European brown bears (Ursus arctos) in south-central Sweden as part of a long term monitoring

Research sample

project. We collected samples from male and female bears of all ages and we used all samples (including adult males, male offspring and non-independent offspring that are still together with their mother) in the supplementary material. There we describe sex-specific population wide patterns of dietary specialization and shifts in diet with age. We there include a total of 554 samples from 115 female and 98 male bears ranging age from 2 - 29 years (median 6 years). It is important to stress that samples taken in spring are representative of the previous years summer diet (i.e. at age 1 - 28). All samples were analyzed for their stable- nitrogen isotopes ($\delta 15N$), stored in bear hair keratin. Stable isotopes reflect cumulative diet intake and are deposited into the hair during growth. We calculated the trophic position of each bear hair sample relative to the average $\delta 15N$ value of moose (mean ± sd = 1.8 ± 1.26 ‰, n = 21, Fig S1). Trophic position is calculated as the discrepancy of $\delta 15N$ in a secondary consumer and its food source divided by the enrichment of $\delta 15N$ per trophic level, plus lambda, the trophic position of the food source (e.g. 1 for primary producers, 2 for primary consumers, 3 for secondary consumer, 4 for tertiary consumers). We used an average trophic enrichment factor of 3.4‰ and added a lambda of 2 given that the moose baseline trophic position is a strict herbivore.
Bear trophic position = (δ15NUrsus arctos – average(δ15NAlces alces)) / 3.4 + 2

Sampling strategy

All datapoint (i.e. samples) with complete information of metadata (age, known mother with representing stable isotope samples, information about home range location after independence, occurrence in the study populations genetic pedigree) were included to obtain the maximum possible sample size. The model is complex in its covariance matrices for genetic pedigree and environmental similarity and we therefore included all available samples.

Data collection

Hair samples were taken during bear captures in spring and early summer (April to June) either during family captures of known females, captures of new bears that were subsequently followed. Bear hair samples were plucked with pliers from between the bears shoulder blades (the person doing this procedure changed over the years but the protocol always remained the same) and stored at the Swedish Veterinary institute. All samples were shipped to the University of Saskachewan where they were washed and grinded in the laboratory of David Janz by Agnieszka Sergiel. The resulting powder was analyzed in the laboratory facilities of Keith Hobson. The first author received the data from the laboratory including information about stable isotope signatures, identity of the bear the sample was taken from and the year the sample was taken. The pedigree was compiled by Shane Frank and published here: https://doi.org/10.1111/eva.13178. Lifetime home ranges were extracted by Jennifer Hansen. Information about familial relationships and age are stored in the database of the Scandinavian brown bear project (responsible: Jonas Kindberg).

Timing and spatial scale

Our data collection includes samples from 1993 until 2016. Given the lag between stable isotope integration and sampling, these samples represent foraging in the years 1992 - 2015.
All samples were collected during bear captures in April - June 1993 - 2016.
Samples were collected from bears in south-central Sweden in the counties of Dalarna and Gävleborg (approximately 250km east-west and 60km north-south in extent), see also Figure of the Study area in the Supplementary Information.

Data exclusions

All female bears with complete information about their age (birth year), maternal identity and at least one maternal stable isotope sample as an adult, home range location after independence, and occurrence in the genetic pedigree where involved in the study shown in the main text. Samples with lacking information on either covariate were excluded. Male bears were excluded because the project does not follow them throughout life but a simpler analysis including male offspring and including the effect of paternal trophic position is presented in the supplementary material. For the model presented in the supplementary material all adult female and male bears with known age information were included. Cubs or yearlings that are still together with the mother were excluded from the main analyses as they are not independently foraging but were included in the supplementary material.

Reproducibility

Our experiment is based on long term data collection and all samples were analyzed as a bulk. The model we are fitting is complex and data hungry precluding the option to fit the model on e.g. two distinct subsets (50%) of data and comparing whether results line up. We have recently received analyzed stable isotope samples from more recent years but our currently available pedigree only spans the period 1990 -2014 and we therefore could neither include those samples into our analysis nor run a separate independent analysis to very our results
Because of the data limitations we have not attempted to repeat the analysis with an independent sample.

Randomization

Randomization was not possible as this study does not involve experimental treatments

Blinding

Lab personnel preparing hair samples and analyzing stable isotopes had no knowledge of the sex, age or family relationships among individual bears (and their samples). Therefore all lab procedures were effectively blinded.

Did the study involve field work? ☒ Yes ☐ No

Field work, collection and transport

Field conditions

Bears were captured in spring and early summer immediately after den emergence. Temperatures were moderate, often with light snow cover remaining from the winter. The Scandinavian brown bear project follows a strict capture and sampling protocol (Arnemo et al., 2006). Field conditions will not affect stable isotope concentrations in bear hair samples because hair is an inert slow growing tissue.

Location

South Central Sweden (ca. 61°N, 15°E), counties of Dalarna and Gävleborg

Access & import/export

Samples were collected and stored in Sweden, then shipped to Poland for preparatory procedures and then shipped to Canada for further analyses. CITES permits were obtained to ship samples. Permitting numbers are: 16PL000376/WP and 15PL000102/WP

Disturbance

To capture bears, bears are darted from a helicopter (see detailed description below under "wild animals"). The SBBRP has captured bears since 1984 and has carried out ca, 3000 captures and immobilizations. It has a capture-related mortality rate of <2% (Arnemo et al., 2006). However, captures are stressful for bears and may impact movement and body temperature for several days after the

capture (unpublished findings). The disturbance by captures would not be expected to bias our data collection, primarily because stable isotopes stored in the hair are representative of the previous year.

Reporting for specific materials, systems and methods

We require information from authors about some types of materials, experimental systems and methods used in many studies. Here, indicate whether each material, system or method listed is relevant to your study. If you are not sure if a list item applies to your research, read the appropriate section before selecting a response.

Materials & experimental systems

n/a	Involved in the study
☒	☐ Antibodies
☒	☐ Eukaryotic cell lines
☒	☐ Palaeontology and archaeology
☐	☒ Animals and other organisms
☒	☐ Clinical data
☒	☐ Dual use research of concern
☒	☐ Plants

Methods

n/a	Involved in the study
☒	☐ ChIP-seq
☒	☐ Flow cytometry
☒	☐ MRI-based neuroimaging

Animals and other research organisms

Policy information about studies involving animals; ARRIVE guidelines recommended for reporting animal research, and Sex and Gender in Research

Laboratory animals	n/a
Wild animals	We captured already collared adult female brown bears (Ursus arctos) and their offspring together in spring to equip offspring with collars and follow them after independence from the mother. We also captured solitary males and females, either as part of a recapture (bears that already carried a collar) or by following tracks in the snow to find new bears. All bears were were darted from a helicopter in spring after den emergence. The time between first sighting of the bear until darting was usually around 15 min and should never exceed 30 min (Arnemo & Fahlman, 2011). After immobilization the bear's eyes were immediately covered to avoid stressful light stimuli and intranasal oxygen supplementation was routinely administered to avoid hypoxemia. Bears were constantly monitored during anesthesia by an experienced team of wildlife veterinarians (Arnemo & Fahlman, 2011). Bears were processed at the location of captures, or in case of family captures (mutiple individuals being darted and processed) occasionally transported to the nearest road access location to meet an extended team of veterenarians. In these cases, bears were transported via helicopter. All bears were sebsequently released. In the case of family captures, all bears were released (i.e., given an antidote to reverse anesthesia) together from the same location.
Reporting on sex	The bulk of our analyses report findings on female bears and their female offspring. The reason for this are a) adult male bears grow much larger and seem to have different dietary compositions and trajectories as adults than female bears. there we would have to fit models individually for each sex. However, male bears disperse out of the study area and are therefore not followed throughout life, precluding the option to track long term effects of maternal learning on male dietary specialization. However, we do provide evidence in the supplementary material that sons indeed initially learn dietary specialization from mothers the same way as daughters do and that the similarity is quantitatively the same for the first 4 years after independence.
Field-collected samples	Hair samples were plucked from between the shoulder blades and stored dry in an envelope at room temperature at the Swedish veterinary institute. Samples were preprocessed (washed, dried and ground to powder) at the Institute of Nature Conservation PAS, Krakow, Poland. They were then shipped to Canada for stable isotope analysis.
Ethics oversight	All animal captures and handling were performed in accordance with relevant guidelines and regulations and were approved by the Swedish authorities and ethical committee (Uppsala Djurförsöksetiska Nämnd: C40/3, C212/9, C47/9, C210/10, C7/12, C268/12, C18/15. Statens Veterinärmediciniska Anstalt, Jordbruksverket, Naturvårdsverket: Dnr 35-846/03, Dnr 412-7093-08 NV, Dnr 412-7327-09 Nv, Dnr 31-11102/12, NV-01758-14). The SBBRP follows a strict and tested capture protocol (Arnemo & Fahlman, 2011) which is approved by the Scandinavian authorities. The SBBRP has captured bears since 1984 and has carried out ca, 3000 captures and immobilizations.It has a capture-related mortality rate of <2% (Arnemo et al., 2006). All capture-related mortalities are reviewed by the State Veterinary Institute of Sweden to establish the cause of death and to improve capture techniques.

Note that full information on the approval of the study protocol must also be provided in the manuscript.

Plants

Seed stocks	n/a
Novel plant genotypes	n/a
Authentication	n/a
